# African swine fever outbreaks in German pig holdings – experiences, epidemiological considerations and genome sequences

**DOI:** 10.1038/s41598-026-36441-1

**Published:** 2026-01-29

**Authors:** Katja Schulz, Sten Calvelage, Lisa Rogoll, Franz J. Conraths, Christoph Staubach, Kerstin Albrecht, Ronny Marquart, Jennifer Kühn, Annett Rudovsky, Dieter Brunklaus, Gerald Stumpf, Jeannine Gruse, Jörn Gethmann, Sandra Blome, Carola Sauter-Louis

**Affiliations:** 1 Friedrich-Loeffler-Institut, Federal Research Institute for Animal Health, Institute of Epidemiology, Greifswald-Insel Riems, 17493 Germany; 2 Friedrich-Loeffler-Institut, Federal Research Institute for Animal Health, Institute of Diagnostic Virology, Greifswald-Insel Riems, 17493 Germany; 3 Friedrich-Loeffler-Institut, Federal Research Institute for Animal Health, Greifswald-Insel Riems, 17493 Germany; 4 State Office for Occupational Safety, Consumer Protection and Health, Department V2 – Animal Disease Prevention and Control, Animal Disease Control Service, 14478 Potsdam, Germany; 5 Department of Veterinary Services and Consumer Protection, Emsland District, 49716 Meppen, Germany; 6 Veterinary and Food Inspection Office, District of Rostock, 18273 Güstrow, Germany; 7 State Office for Agriculture, Food Safety and Fisheries of Mecklenburg-Western Pomerania, 18059 Rostock, Germany

**Keywords:** African swine fever, Outbreak investigations, Genome sequencing, Domestic pigs, Wild boar, Epidemiology, Computational biology and bioinformatics, Diseases, Genetics, Microbiology

## Abstract

Until December 2025, 18 African swine fever outbreaks have occurred in domestic pig holdings in Germany. However, nine of them emerged in western Germany in 2024, representing a separate spatial and temporal cluster. Thus, the current study is limited to the remaining nine outbreaks. We aimed to illustrate the epidemiological background of each of the nine outbreaks. The assessments included the results of the outbreak investigations and of the genome sequencing. This is the first study to provide a detailed overview of these outbreaks and, in particular, to publish the genome sequences involved. In several outbreaks, a connection to the neighboring affected wild boar population was considered likely. The hypotheses were supported by genome sequencing; thus, the study has underscored the benefit of including results of genome sequencing in the evaluation of disease epidemiology. However, outbreaks also occurred on farms without reported cases in wild boar in the vicinity. Similarly, outbreaks were observed in farms with low biosecurity standards but also in farms with very high biosecurity, emphasizing the difficulty to identify a pattern in the outbreaks. Still, it has to be acknowledged that a consistent and sustained compliance with biosecurity measures represents the most important factor in preventing virus introduction.

**Supplementary Information:**

The online version contains supplementary material available at 10.1038/s41598-026-36441-1.

## Introduction

After its introduction into Georgia in 2007, African swine fever virus (ASFV) genotype II spread in the Caucasus region and in Eastern Europe, before it emerged in the European Union (EU) in 2014. The first countries affected were Lithuania and Poland, closely followed by Latvia and Estonia^1–4^. In the meantime, however, many more countries within the EU, but also beyond, are affected^5^. The animal disease has also spread to large parts of Asia and to the Caribbean^5^. Germany reported the first detection of ASFV-positive wild boar in September 2020^6^.

In the current epidemic in Europe, wild boar are the most affected species, contributing significantly to the spread of the disease^7,8^. Nevertheless, virus introduction into domestic pig herds also occurred sooner or later in the majority of affected European countries^9^.

A consistent picture regarding the characteristics of African swine fever (ASF) outbreaks in farms keeping domestic pigs including the accompanying risk factors is lacking. In many studies, the presence of an ASFV-infected wild boar population near the outbreak farm has been considered as a risk factor for ASFV introduction into domestic pigs^1,10–12^. Insufficient biosecurity and management measures have also been identified as a potential cause of virus introduction^12–16^. Such inadequate management is frequently associated with smaller, non-industrial pig-holdings, which often house less than 10 pigs or keep their animals outdoor^9,12^. Yet, also huge pig farms experienced ASF outbreaks, suggesting that human activities, farm management practices and gaps in or violations of biosafety protocols played a role in ASFV introduction.

Although several hypotheses related to risk factors and virus entry pathways exist in different countries^12,17–19^, it is challenging to identify the definitive source of introduction for each outbreak^16^. Epidemiological and virological analyses are commonly applied to identify the most likely entry point of each outbreak. Moreover, serological testing for ASFV-specific antibodies is recommended to estimate the approximate time of introduction and to gain a clearer understanding of disease progression within the farm. The results of these analyses may also indicate point introductions or a broader introduction of contaminated material to several animals or units in the holding. A clear picture of the virus spread within the farm facilitates tracing back and tracing forward and can thus help to minimize further disease spread. However, beside its usefulness, serological testing does not replace passive surveillance, which should be maintained to detect virus introduction as early as possible.

Over the past decades, next-generation sequencing (NGS) has become a powerful tool in molecular diagnostics enabling the identification and full genetic characterization of pathogens. The capability of discriminating circulating ASFV strains in Germany by whole genome sequencing was demonstrated in a previous study^20^. It revealed the emergence of several new mutations in the ASFV genome and furthermore showed the geographic clustering of genetically closely related ASFV variants. Based on these findings, whole genome sequencing can provide indications for geographical links between ASF outbreaks by the comparison of mutation profiles of known and novel virus variant genomes. However, NGS is limited by its restricted availability and application, which may also lead to potentially premature interpretations concerning epidemiological associations. Yet, whole-genome sequencing plays an important role in epidemiological outbreak investigations and should be used to enhance conventional approaches wherever possible.

**Fig. 1:**
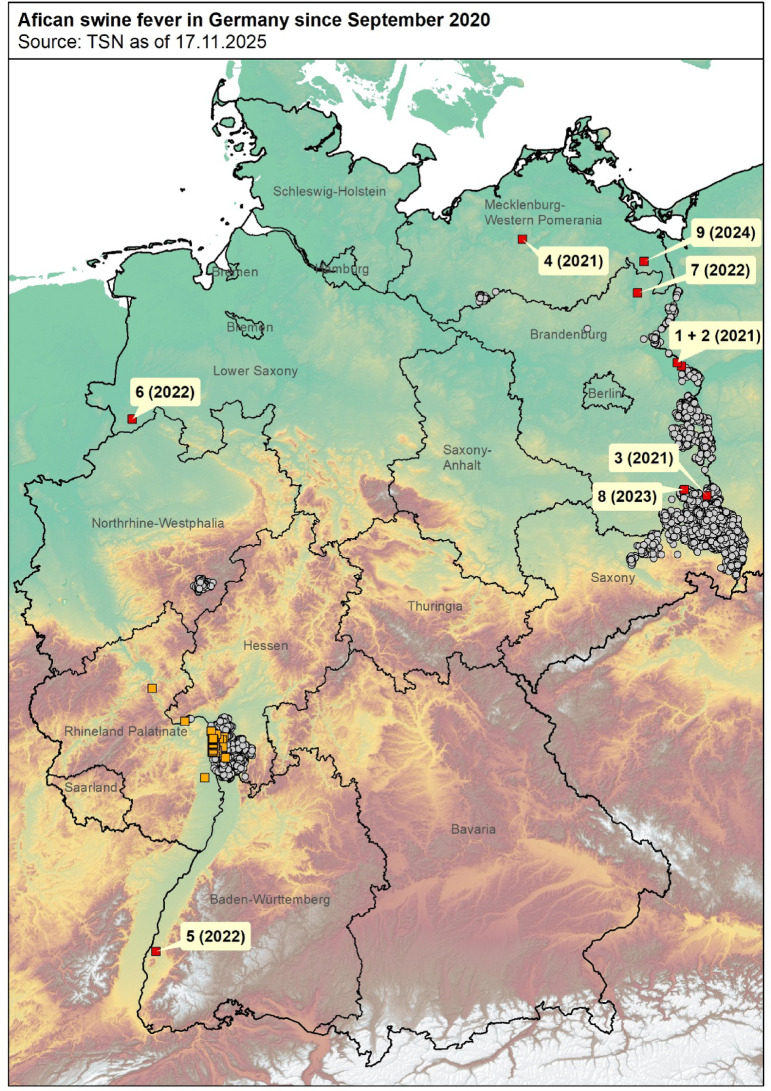
Cases of ASF in wild boar and outbreaks in domestic pigs in Germany from 10th September 2020 until the 17th November 2025. Grey dots represent ASF cases in wild boar, red squares ASF outbreaks in domestic pig farms. Orange squares represent the cases in the Hesse and Rhineland-Palatinate cluster. Numbers represent the number of each individual outbreak as listed in the manuscript including the year of the outbreak. Map was generated by using ArcGIS ArcMap 10.8.2 (ESRI, Redlands, CA, USA).

In the present paper, we describe the epidemiological situation of ASF in German domestic pig holdings. Until December 2025, 18 outbreaks occurred in six of the 16 German federal states (one additional outbreak in a wild boar holding). These outbreaks differed in their dimensions, e.g. regarding farm size and ASF-affected pigs, and their epidemiological characteristics. Also, the majority of them were isolated, independent outbreaks. In contrast, a cluster of nine outbreaks that occurred in 2024 in Hesse and Rhineland-Palatinate (Fig. 1) differed from the other nine outbreaks and, therefore, will not be included in this study.

The description and epidemiological appraisal of the German ASF outbreaks may help to deal with similar situations in other countries and facilitate a joint approach to control ASF at the international level.

## Materials and methods

In this paper, we describe the outbreaks in a systematic way. We started by presenting background information on each outbreak, followed by a detailed account of key findings from the epidemiological investigations and genome sequencing analyses. Finally, we synthesized these results into a summary and a conclusion on the course of the disease in the affected premises and the most likely source(s) of ASFV introduction.

### Epidemiological investigations

Germany consists of 16 federal states. Animal disease control in Germany is organized in a decentralized manner and is the responsibility of the individual federal states.

Following the confirmation of an ASF outbreak, the competent authorities the affected federal state carry out an epidemiological outbreak investigation, with support from epidemiologists of the Federal Research Institute for Animal Health, the Friedrich-Loeffler-Institut (FLI), provided upon request by the federal state. The investigation primarily aims at addressing the following questions: when was the virus introduced into the farm (time of entry), and what was the route of introduction? In addition, backward and forward tracing are conducted to minimize the risk of further disease spread.

### Laboratory diagnostics

In Germany, the standard procedure for diagnosing ASF begins with initial testing at a state laboratory accredited according to ISO/IEC 17025 within the affected federal state. These laboratories regularly participate in inter-laboratory comparison tests conducted by the National Reference Laboratory (NRL). Samples yielding positive or inconclusive results in the respective competent state laboratory of the affected federal state are immediately submitted to the NRL for confirmatory testing, as only an NRL diagnosis allows to confirm an outbreak or case. At the NRL, licensed commercial qPCR kits—primarily the virotype ASFV 2.0 kit (Indical Bioscience, Leipzig, Germany)—are used following manual or automated DNA extraction (QIAamp Viral RNA Mini kit, [Qiagen, Hilden, Germany] or NucleoMagVet kit [Macherey-Nagel, Düren, Germany] on a KingFisher platform [Thermo Fisher Scientific, Waltham, USA).

To isolate ASFV, a haemadsorption test (HAT) is performed on PBMC-derived macrophages using a slightly modified standard procedure^21^. Virus isolates are archived for further in vitro and in vivo testing.

### DNA extraction for sequencing

DNA extraction was performed with the NucleoMagVet kit (Macherey-Nagel) according to manufacturer instructions. For samples from outbreak farm 9, lysis was achieved following the first steps of the NulceoMagVet kit before the lysate was further processed with the RNAdvance Tissue Kit (Beckmann Coulter, Brea, USA). Obtained DNAs were quantified and checked for impurities on an Implen N60 NanoPhotometer (Implen GmbH, Munich, Germany).

### Sequencing (Sanger and NGS)

In order to generate reliable whole-genome sequences of ASFV strains a combination of sequencing techniques and platforms was chosen according to the requirements of each investigated sample set. An overview of platforms and techniques used for each sample is given in (Table 1).

**Table 1:** List of analysed samples from domestic pig outbreaks and genetically related wild Boar cases. Sequencing strategies are specified in the corresponding materials and methods section.

ASFV domestic pig outbreaks in Germany										
Outbreak farm	Location	Animal sample	Lib-no.	Sample matrix	Sequencing strategy	Mean coverage [reads/site]*	Reads ASFV*	Genome length [bp]	Comments	Accession
1, 2	Brandenburg	2021ASP03384	lib05117	EDTA blood	Illumina	202	260.256	190.517	Complete genome published in previous study^20^	OX376265
3	Brandenburg	2021ASP03286	lib05058 + lib05059	Blood swap	Enrichment + Illumina / Amplicon Sanger sequencing	-	524 (Illumina) 3 (Sanger)	n.d.	Only partial sequences available (Sanger)	
4	Mecklenburg-Western Pomerania	2021ASP05548	lib05327	Organ pool	Enrichment + Illumina	222	282.069	190.591	Low coverage of EP402R gene	OZ335133
5	Baden-Wuerttemberg	2022ASP01802	lib05910	EDTA blood	Illumina	213	280.531	190.598	No German genetic markers identified	OZ335129
6	Lower Saxony	2022ASP01931	lib05777	EDTA blood	Illumina	244	313.365	190.609		OZ335128
7	Brandenburg	2022ASP01938	lib05778	Lung tissue	Illumina	53	68.560	190.602		OZ335136
8	Brandenburg	2023ASP00680 2023ASP00683	lib06111-12 + lib06119-22	Lung + spleen tissue	Enrichment + Illumina	28	36.451	190.571		OZ335137
9	Mecklenburg-Western Pomerania	2024ASP01428	lib06609 (Nanopore) lib06605 (Illumina)	Spleen tissue	Nanopore + Illumina	9 (Nanopore) 423 (Illumina)	2.239 (Nanopore) 541.829 (Illumina)	190.621		OZ335132

ASFV wild boar cases genetically related to outbreaks in domestic pigs										
Related outbreak farms	Location	Animal sample	Lib-No.	Sample matrix	Sequencing strategy	Mean coverage [reads/site]*	Reads ASFV*	Genome length [bp]	Loci of shared characteristic mutations	
6, 7, 9	Brandenburg	2022ASP01516	lib05907	EDTA blood	Illumina	20	25.402	190.591	MGF 505-6R	OZ335138
8	Brandenburg	2022ASP03250	lib05995	EDTA blood	Illumina	48	63.186	190.597	MGF 505-2R, EP424R, M448R, I267L	OZ335135
8	Brandenburg	2022ASP03254	lib05996	EDTA blood	Illumina	18	22.824	190.569	MGF 505-2R, EP424R, M448R, I267L	OZ335134
8	Brandenburg	2022ASP03260	Lib05997	EDTA blood	Illumina	160	211.707	190.593	MGF 505-2R, EP424R, M448R, I267L, MGF 360-21R	OZ335130
6, 7, 9	Brandenburg	2024ASP06001	lib06964 (Nanopore) lib07083 (Illumina)	Tonsil	Nanopore + Illumina	4 (Nanopore) 75 (Illumina)	1.202 (Nanopore) 96.119 (Illumina)	190.574	MGF 505-6R	OZ335131

*Mean Coverage and Reads ASFV were determined after a mapping of the complete dataset against the reference sequence ASFV Germany 2020/21 (LR899193).

Methods used for the generation of Illumina and Sanger sequencing datasets were described elsewhere^20^. In brief, samples were sequenced either on an iSeq 100 platform (Illumina, San Diego, USA) in combination with the GeneRead DNA Library I Core Kit (Qiagen) and Netflex Dual-index DNA Barcodes (PerkinElmer, Shelton, USA) or, to scale up data volume in a cost-effective way, send to Eurofins (Eurofins Genomics Europe Shared Services GmbH, Ebersberg, Germany), a commercial sequencing service that utilizes a NovaSeq 6000 platform (Illumina) and a modified NEBNext Ultra™ II FS DNA library preparation (New England Biolabs, Ipswich, USA). For the latter, a minimum of 100 ng DNA was send for sequencing.

Sanger sequencing was conducted to allow the classification of viruses from outbreak farm 3. Gene regions covering D339L, K196R and MGF 360-15R were selected for amplification according to experiences of potential virus variants and their mutation profile occurring in affected regions^20^. Therefore, previously described and evaluated primer pairs^20^ were used for the generation of amplicons which. were subsequently sent to Microsynth Seqlab GmbH (Göttingen, Germany) for sequencing.

Nanopore sequencing was conducted on a P2 solo platform (Oxford Nanopore Technologies, Oxford, UK) and PromethION Flow Cells (R10.4.1 FLO-PRO114M, Oxford Nanopore Technologies). Library construction was performed utilizing the Rapid Barcoding Kit (SQK-RBK114.24, Oxford Nanopore Technologies) according to manufacturer recommendations with the following adaptations: pools of barcoded libraries were eluted twice in 15 µL elution buffer during the bead-based clean-up. To the 30 µL total volume of cleaned pool, 2.5 µL of the mixture of rapid adapter and adapter buffer were added for a final pool volume of 32.5 µL. Sequencing was conducted for 72 h in super accurate basecalling mode with barcode trim enabled.

### Data analysis

Reads generated by NGS were mapped along the German ASFV reference sequence ASFV Germany 2020/21 (LR899193)^6^ using either Newbler 3.0 (Roche, Basel, Switzerland) with default parameters (including quality and adapter trimming) for Illumina-derived reads or Minimap2 v2.24^22^ with default parameters for nanopore reads. Subsequently, mapping results were visualized in Geneious Prime 2021.0.1 (build 2020-12-01, Dotmatics, Boston, USA) and differences between the reference sequence ASFV Germany 2020/21 (LR899193) and mapped reads manually inspected. Identified mutations were listed according to HGVS nomenclature^23^, for which genome positions were reported in reference to ASFV Georgia 2007/1 (FR682468.2) prior to positions in ASFV Germany 2020/21 (LR899193) due to the broader application of ASFV Georgia 2007/1 (FR682468.2) in the literature. To determine the closest genetic relatives of the ASFV strain from outbreak farm 5, the assembled viral genome was subjected to a BLASTn Megablast analysis using default parameters against the NCBI core nucleotide database (National Center for Biotechnology Information, Bethesda, USA)^24^.

The assembled whole-genomes were submitted to the European Nucleotide Archive (ENA, European Bioinformatics Institute, Hinxton, UK) under the project accession PRJEB89459.

Enrichment of ASFV-related library molecules was realized under usage of the hybridization capturing kit from Daicel (Osaka, Japan). This kit provided a customized biotinylated RNA oligomer set (baits) that was specifically designed based on a selection of 39 available ASFV whole-genome sequences representing the majority of known ASFV genotypes. Capturing was conducted according to steps listed in the manufacturer’s manual (v5.02) and as described by Wylezich, et al.^25^ with a hybridization temperature set to 63 °C and 14 (outbreak 3 and 8) or 18 (outbreak farm 4) cycles of amplification after capturing. Subsequently, enriched libraries were sequenced on the iSeq 100 as described above and by Forth, et al.^20^.

## Results

In July 2021, the first three ASF outbreaks in domestic pig holdings were reported in Germany in the federal state Brandenburg (Fig. 1).

### Outbreak farm 1 and 2 (Brandenburg)

#### Background information

Mid-July 2021, the first two ASF outbreaks in domestic pig holdings were confirmed. The small backyard farms with only two (farm 1) and four fattening pigs (farm 2), respectively, were located in the district Märkisch-Oderland. The pigs were kept for private consumption. The two affected holdings were in a linear distance of 4.6 km apart from each other and no more than 3 km away from the German-Polish border. In the affected municipality, several ASF-positive wild boar had been detected in the months before the outbreaks. The outbreak farms were thus located in a restriction zone. On both farms, other animals, like dogs or poultry were kept. According to the owners, the pigs were kept indoors, but at least one of them also had an outdoor run that had obviously been used by pigs shortly before the outbreak occurred. The biosecurity measures on both private holdings were marginal to non-existent.

#### Course of events

Clinical signs first occurred on farm 1. They had started several days before ASF was suspected and then officially reported. The animals were first treated by a local veterinarian who first suspected a bacterial disease. Only after the treatment attempt had remained unsuccessful, ASF was suspected. On farm 2, clinical signs only occurred one day before the ASF diagnosis. In both holdings, all pigs were tested positive for ASFV. In addition, ASFV-specific antibodies were detected in farm 1 in one of the pigs. In farm 2, no ASFV-specific antibodies were detected.

#### Epidemiological investigations

In both backyard farms, different ways of indirect virus introduction were considered possible. Feed (e.g. grass, at least in one outbreak) and/or bedding material had been introduced, which may have been contaminated with ASFV. The owner of farm 1 was a hunter. He had been engaged in outdoor activities in the area surrounding the farm shortly before the outbreak occurred. Human activities resulting from a lack of compliance regarding basic biosecurity measures may also have been the cause of ASFV introduction.

#### Genome sequencing

The genetic characterization of the causative ASFV variant from farms 1 and 2 was conducted as part of an early German ASFV surveillance program, which was systematically evaluated and presented in a previous publication^20^. To retain a consistent report structure of all presented domestic pig outbreaks, a detailed description of the results was also added for this outbreak: EDTA blood samples were chosen for NGS library preparation and sequenced as previously reported^20^. In summary, highest viral read counts were obtained from an EDTA blood sample (2021ASP03384; ct 19) with 260,256 viral reads (lib05117; mean coverage = 202 reads/site) that resulted in the reconstruction of the whole virus genome. The comparison of the obtained consensus sequence with ASFV Germany 2020/21 (LR899193) revealed three mutations: a nucleotide exchange in the MGF 360–10 L gene (c.27.197G > A) leading to a nonsense mutation as well as a deletion in the MGF 505-4R (c.37.027del) and MGF 100–3 L (c.181.407del), respectively. All of these mutations have a high impact on the affected genes as all of them lead to a truncation of the open reading frame. Their joint occurrence was previously reported in German wild boar^20^ forming a lineage of genetically related ASFV variants. According to this classification, the identified virus belongs to Lineage III (see also reference sequence 2021ASP00921 [OX376262] and Figure S1), which is in line with previous sequencing results that confirmed the same characteristic marker mutations in ASFV-positive wild boar from the same time period and district^20^.

#### Conclusions

Considering the clinical course, it can be assumed that all animals had been infected relatively simultaneously from the same source. This makes contaminated feed or bedding the most likely introduction route. In addition, the genetic analysis of the virus genome supports the assumption of the direct or indirect introduction of regionally circulating virus variants from a wild boar source into domestic pigs that might have been fostered by a high virus load in the surrounding environment and low biosecurity standards in the affected pig holdings. The uncertainty of this assessment is low.

### Outbreak farm 3 (Brandenburg)

#### Background information

**Fig. 2:**
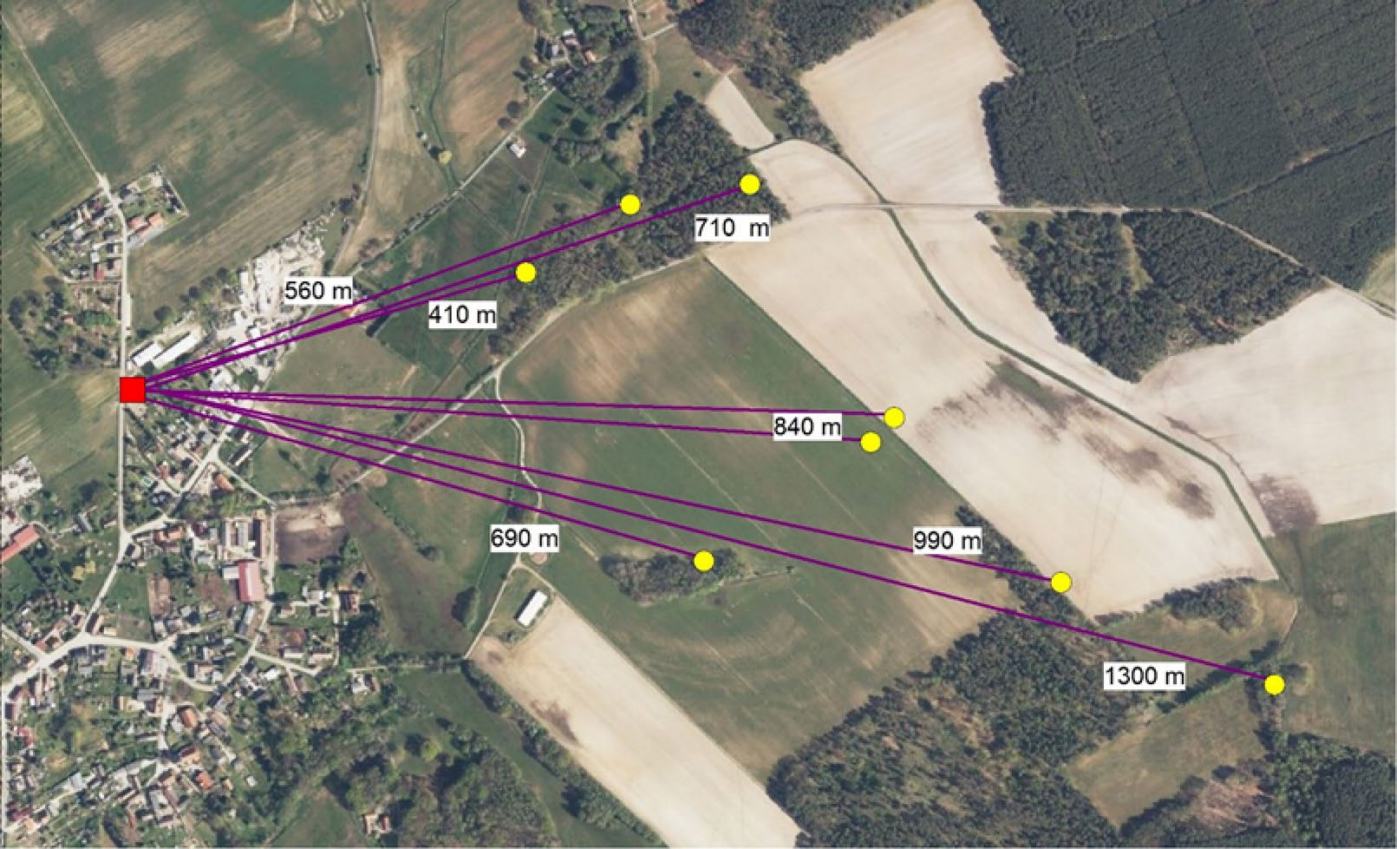
ASFV-positive wild boar carcasses (yellow dots) in the east of the outbreak farm (red rectangle). Distances to the farm are provided in meters (m). Map: data licence Germany – attribution – version 2.0 (https://www.govdata.de/dl-de/by-2-0).

On the same day when samples from the first two outbreaks were tested positive for ASFV, another outbreak was officially reported appr. 135 km south. The affected farm was located in a forestry area in the district Spree-Neiße. Almost one year before, the first ASF-infected German wild boar had been detected in this district (appr. 43 km distance) and several ASFV-positive wild boar had been detected in the vicinity of the farm shortly before and at the time of the outbreak. Many of the detection sites of the positive carcasses were less than one km away (Fig. 2).

The affected piggery was an organic breeding farm following the regulations of Biopark^®^ (https://biopark.de/wp-content/uploads/2021/07/Erzeuger_Richtlinie_Biopark.pdf), presenting reasonable biosecurity measures. At the time of the outbreak, appr. 300 pigs were kept on the farm. These animals were divided between two stables, depending on their age and pregnancy status. Due to the ASF situation in wild boar, all animals were kept indoors. However, due to the lack of an artificial ventilation, there were open doors and windows; thus, contact to the environment was possible. The farm was completely fenced with different fences like wire mesh and electric fences. However, not all areas were double-fenced.

#### Course of events

Two days after the onset of clinical signs like cyanotic skin alterations and reduced feed and water intake, one breeding sow died. This animal subsequently tested positive for ASFV. From 61 analysed samples, only one additional animal housed in the same pen tested positive for the virus in subsequent investigations. All samples were tested negative for ASFV-specific antibodies, indicating that the disease had been detected at a very early stage.

#### Epidemiological investigations

The results of the epidemiological investigations remained inconclusive regarding the introduction route. However, it was considered unlikely that the virus was introduced by persons or vehicles. Indirect virus introduction through contaminated shoes or other animals was conceivable, particularly due to the high infection pressure by ASF in wild boar in the surroundings. The pen in which the infected sows were housed was the only one with a door to the outside. In addition, this door was usually open to secure ventilation and only closed with a gate.

#### Genome sequencing

Sample material from outbreak farm three was limited to blood swabs, which yielded only low quantities of DNA after nucleic acid extraction. An initial sequencing run performed on an iSEQ 100 platform produced 300 ASFV reads, which was insufficient for subsequent analyses. Accordingly, an enrichment of virus-derived library molecules was performed by a hybridization capturing approach that utilizes a designed library of ASFV sequence-specific, biotinylated oligomers. However, the enriched library returned only small numbers of total reads and added only 224 more ASFV reads to the dataset. In order to enable a reliable classification of the virus variant while overcoming the limited availability of DNA, an amplicon-based Sanger sequencing approach was conducted to generate partial gene sequences of the genes D339L, K196R and MGF 360-15R as described elsewhere^20^. The sequences of the amplicons were aligned to ASFV Germany 2020/21 (LR899193) and revealed a base exchange in the MGF 360-15R gene (g.50.922 C > T) leading to a nonsense mutation and consequently to the truncation of the open reading frame. This mutation was previously described as genetic marker for ASF virus variants classified as Lineage IV (see also reference sequence 2020ASP02805 - OX376250)^20^. Further subdivisions of this lineage showed mutations, inter alia, in the D339L (g.140.696G > A) and K196R gene (g.65.259 C > T), however, the Sanger results showed no mutations in these particular genetic regions. This is in accordance with reports about circulating ASFV variants in wild boar in the district Spree-Neiße at that time, as all of them were identified as Lineage IV members without mutations in D339L and K196R.

#### Conclusions

Based on the epidemiological findings including the results from the genome sequencing, indirect virus introduction from the infected wild boar population in the immediate vicinity was considered the most likely route of introduction. The uncertainty of this assessment is low.

### Outbreak farm 4 (Mecklenburg-Western Pomerania)

#### Background information

Until November 2021, the federal state of Mecklenburg-Western Pomerania was free from ASF in both, wild boar and domestic pigs. However, in November 2021, an outbreak occurred in a fattening farm in the district Rostock located approx. 100 km west to the Polish border and more than 200 km away from the outbreaks in Brandenburg. The farm, surrounded by agricultural land, was a conventional fattening farm with appr. 4,000 pigs. The whole premises were fenced and inaccessible to unauthorised persons. The animals were housed in two separate stables, subdivided into compartments and pens.

The general biosecurity measures seemed sufficient. The pigs were fed with industrially produced feed and had no bedding material. Several people handling the pigs stated that they were active hunters and at least some admitted to having engaged in hunting activities in ASF-affected areas.

#### Course of events

Five days before the official confirmation of ASF, clinical signs like fever occurred in one compartment, followed by increased mortality. Sick animals were unsuccessfully treated with antibiotics and nonsteroidal anti-inflammatory drugs. Animals from this compartment subsequently tested positive for ASFV. Only in one additional compartment, further animals were affected. No testing for ASFV-specific antibodies was performed.

#### Epidemiological investigations

Disease transmission within the compartment may be explained by multiple use of cannulas in the treatment of animals. In addition, shoes were not changed after inspecting the pigs that were the first to fall ill. Virus introduction through feed, bedding or other animals was considered unlikely, while hunting activities of persons who care for pigs may generally represent a high risk, particularly if they visit areas with an ASF-infected wild population.

#### Genome sequencing

The obtained sequencing data resulted in a small number of viral reads and a high portion of host genomic background, which provided only a fragmentary coverage of the ASFV genome. Like for samples from outbreak farm three, a hybridization capturing approach was utilized to modulate the virus/host-ratio in favour of the viral sequences. Sequencing of the enriched library resulted in a total of 282.069 viral reads and a mean coverage of the whole ASFV genome of 222 reads/site, however, coverage was significantly reduced in a small gene region of EP402R of approx. 500 nt length and thus excluded from further analyses. The comparison of the assembled genome with ASFV Germany 2020/21 (LR899193) revealed characteristic mutations in the genes MGF 360–10 L, MGF 505-4R and MGF 100–3 L that correspond to the described mutations defined for ASFV Lineage III (Forth, et al.^20^, outbreak farm 1 and 2, Figure S1). Moreover, a so far unknown mutation could be observed in the I329L gene (g.173.628T > A) leading to the amino acid change His293Leu. This mutation was also later found in a deceased wild boar located approx. 60 km southeast from the affected facility and could be confirmed for at least 19 more cases between November 2021 and May 2022 from this region by a tailored Sanger sequencing approach. Despite its predominant distribution in the affected region at that time, the I329L mutation could not be detected in any other ASF outbreaks in Germany, supporting the scenario of a point introduction from e.g. western Poland representing the closest external outbreak area for which only limited sequencing information are available yet.

#### Conclusions

A definite route of virus introduction could not be determined. Yet, indirect introduction due to a lack of attention to strict compliance with biosecurity measures seemed conceivable. The uncertainty of this assessment is moderate.

### Outbreak farm 5 (Baden-Württemberg)

#### Background information

Baden-Wuerttemberg had been historically free from ASF in both domestic pigs and wild boar before the first outbreak in domestic pigs in May 2022. Baden-Wuerttemberg is located in south-west Germany and thus, far away from the previous ASF events in Germany and elsewhere in Europe (Fig. 1).

The holding was an outdoor fattening farm. At the time of the outbreak, 19 pigs were kept on a triple fenced field, few kilometres away from the main farm premises, but only 250 m away from the forest. As fruit and vegetables are main products of the farm, several seasonal harvest workers from south-east European countries were employed at the time of the outbreak.

#### Course of events

The first pig died four days before the official confirmation of an ASF-outbreak. Over a course of a few days, the mortality increased dramatically. All culled animals tested ASFV-positive, but negative for ASFV-specific antibodies. Thus, it is very likely that all animals had come into contact with the virus at almost the same time.

#### Epidemiological investigations

Neither the farm owner nor any of the employees were engaged in hunting activities. Moreover, there was no indication that mechanical vectors (vehicles, shared tools etc.) might have played a role in virus introduction. Intensive carcass search in the surrounding area of the farm and ASF-testing in wild boar did not indicate virus circulation within the wild boar population in Baden-Wuerttemberg. In addition, a direct wild boar-pig contact was considered hardly possible due to the triple fencing. Thus, the probability of a direct or indirect virus introduction through wild boar was deemed negligible.

Yet, the pigs were, among others, fed with the fruit and vegetable waste, which could theoretically have been contaminated with ASFV-contaminated food.

#### Genome sequencing

Sequencing was performed on multiple samples with different viral loads based on availability of sample material. Highest viral read numbers were obtained for an EDTA blood sample (lib05910; 2022ASP01802) that resulted in 280,531 ASFV reads covering the whole virus genome (mean coverage = 213 reads/site). The comparison of the assembled genome with ASFV Germany 2020/21 (LR899193) revealed nine differences in non-homopolymer regions whereas none of the previously reported mutations found in German cases could be detected^20^ (Supplemental material Table S1). Moreover, a 14 nt long insertion in the O174L gene, a key mutation common to all German ASFV variants at the time, was absent, making the introduction from the known outbreak regions in eastern Germany highly unlikely. This is further highlighted in the conducted phylogenetic tree, as the sequence clearly separates from the clusters of the lineages defined for German ASFV variants (Figure S1). To elucidate the potential origin of the ASFV variant, a blastn analysis was conducted. The results revealed the highest genetic similarity of the Baden-Wuerttemberg sequence with an ASFV sequence from the Republic of Moldova (ASFV Moldova 2017/1 [LR722599.1]) as well as two Italian sequences from 2022 (20355/RM/2022 [OP605386.1]^26^ and 21730_1474/RM/2022 [OR460730.1]).

#### Conclusions

Genome sequencing indicated that the virus that caused outbreak 5 was not related to the ones observed in the wild boar affected areas of Eastern Germany. These findings and the indication of absence of ASF in wild boar in the region further support the hypothesis of an introduction of the identified virus variant by an anthropogenic source from a country that was affected by active ASF outbreaks in 2022, e.g. through seasonal harvest workers or friends or relatives visiting them from countries in south-eastern Europe. The uncertainty of this assessment is moderate.

### Outbreak farm 6 (Lower Saxony)

#### Background information

End of June 2022, similar to the outbreak in Baden-Wuerttemberg, an isolated ASF-outbreak was reported from the federal state of Lower Saxony (Fig. 2). The piggery was a conventional farm with breeding pigs (294 sows, 469 piglets) and piglet rearing (1,067 piglets)^27^. The farm premises were fenced, only accessible through a sliding gate and all buildings were in a good shape. The breeding animals and the rearing piglets were kept in two completely separated stables. Farm biosecurity and general hygiene measures were considered good.

#### Course of events

Almost one week before the official declaration of the ASF outbreak, two sows from the same compartment had died peracutely. After two more animals had died and few more showed clinical signs, the group was treated with antibiotics without any improvement. Subsequently, two more sows within the same compartment showed clinical signs of infection and thus, samples were taken for ASF diagnostics. These two sows tested positive for ASFV. Furthermore, in the course of the depopulation measures seven PCR-positive animals were detected in the breeding facility, following the relocation of diseased sows from the primarily infected waiting area to the breeding facility. Following the temporary relocation from the infected waiting area to the farrowing unit, one further sow was tested ASFV-positive. No testing for ASFV-specific antibodies was performed.

#### Epidemiological investigations

No clear result regarding the route of virus introduction has emerged from the epidemiological investigations. Direct virus introduction through potential pig purchase was unlikely as all pig movements did not match the observed course of the disease and the corresponding estimated time of virus introduction. Also, direct contact with wild boar has been ruled out as source of virus introduction due to the high biosecurity measures and the absence of the virus within the local wild boar population. Reduced hunting activities, low personnel movements and again high biosecurity measures made various routes only theoretically possible.

#### Genome sequencing

EDTA blood and organ pools of infected animals were available for sequencing and samples with the highest viral load were further processed. The sequencing of the selected samples yielded sufficient reads for in-depth analysis with peak ASFV read counts of 313,365 (mean coverage = 244 reads/site) for an EDTA blood sample tested positive with a cycle threshold (ct) value of 16 by qPCR (lib05777; 2022ASP01931). The comparison of the whole genome with the German reference enabled the classification of the virus variant as member of the Lineage III (mutations: MGF 360–10 L, MGF 505-4R and MGF 100–3 L), but also revealed four distinct differences that were never described before. Of these, two represent extensions of homopolymers in intergenic regions (g.24.994_24.995insA; g.187.978_187.979insA), one base exchange in an intergenic region (g.17.599T > A), and one base exchange in the coding region of MGF 505-6R (g.41.409G > A; p.Cys465Tyr) (Supplemental material Table S1). The latter was also found in a deceased wild boar in the north-east of the federal state of Brandenburg in 2022 (district Uckermark; 2022ASP01516, lib05907, Table 1) and reappeared in 2024 in a virus variant affecting a domestic pig holding in the south-east of Mecklenburg-Western Pomerania (outbreak farm 9). Later, it was again found in a wild boar in the north of Brandenburg (district Oberhavel; 2024ASP06001, lib07083, Table 1). Moreover, this MGF 505-6R mutation was also determined in samples collected from outbreak farm 7, which reported an ASFV outbreak shortly after the confirmation of the onset of ASF in Lower Saxony. The high genetic relationship of these ASFV variants is also reflected in the phylogenetic analysis, as they form a group in the cluster of Lineage III (Figure S1).

#### Conclusions

Considering the whole picture, a virus introduction through trade still seems to be the most likely cause of virus introduction, as there were indirect connections with farms in Brandenburg (particularly to outbreak farm 7). The uncertainty of this assessment is high.

### Outbreak farm 7 (Brandenburg)

#### Background information

Almost simultaneously with the outbreak in Lower Saxony, the federal state of Brandenburg reported an ASF outbreak in June 2022 on a farm with approx. 1,038 fattening pigs. The outbreak farm was located appr. 100 km north of the two first outbreaks in 2021 (Fig. 1) and 30 km away from the closest ASF-positive case in wild boar (district Uckermark). Thus, the farm was not located in the restriction zone established at that time. Farm biosecurity and general hygiene measures were considered good. The farm was surrounded by fields. The owner also operated a large breeding facility at a distance of about 500 m.

#### Course of events

In one compartment of the fattening farm, the pigs were treated for different suspected diseases and several animals had died over the course of two months. In June 2022, two animals of one compartment tested positive for ASFV. After the official declaration of the outbreak, further samples were investigated. Within the affected compartment, all animals were sampled. Twenty-two of 42 samples tested positive for ASFV. Thirty-nine of these samples tested positive for antibodies; accordingly, 17 samples from this compartment were antibody-positive but virus-negative, suggesting that ASFV introduction had taken place already long before the outbreak was officially confirmed. From the other compartments on the fattening farm and from the breeding unit, a representative sample size, following the “Leitfaden zur Bestimmung von Stichprobenumfängen” (Guideline for Determining Sample Sizes) of the Friedrich-Loeffler-Institut (https://www.openagrar.de/servlets/MCRFileNodeServlet/openagrar_derivate_00033966/Stichprobenuntersuchungen_20201119_final.pdf) was taken. Accordingly, from each unit, 60 samples were investigated and subsequently tested negative for ASFV and ASFV-specific antibodies.

#### Epidemiological investigations

The epidemiological investigations failed to disclose any unambiguous introduction route. No virus was detected in the wild boar population in the close vicinity of the outbreak. In the stables, no bedding was used and all pigs originated from the breeding farm of the same owner. None of the personnel handling domestic pigs reported contact to wild boar or other pigs, making a direct or indirect virus introduction through vectors unlikely. Yet, the time of ASFV introduction must have been at least few months before the confirmation of the disease, which may also explain the number of dead animals in this compartment, but increases the difficulty in identifying conclusive evidence for the route of virus introduction.

#### Genome sequencing

A broad range of sample matrices was available from infected animals including tissue samples from lungs and lymph nodes as well as EDTA blood. Sequencing of selected samples returned highest viral read counts for lib05778 (68,560 reads, mean coverage = 53 reads/site) that was prepared from DNA extracted from lung material. The analysis of the obtained ASFV genome presented a similar mutation pattern as found in the outbreak in Lower Saxony, i.e. the markers for Lineage III and the mutations g.17.599T > A, g.24.994_24.995insA and the MGF 505-6R substitution g.41.409G > A (p.Cys465Tyr). However, two variations between both viral genomes were identified: the absence of the homopolymer extension g.187.978_187.979insA in outbreak farm 7 compared to 6, and an additional, hitherto unknown substitution in the EP424R gene (g.72.358G > T; p.Gly255Cys) in the sample from outbreak farm 7.

#### Conclusions

Considering the ASFV-negative results on the breeding facility, it is considered unlikely that diseased animals entered the fattening farm. Possibly, an indirect introduction due to a lack of attention to strict compliance with biosecurity measures may be conceivable on the fattening farm. While the short chronological interval and the relatedness of sequences may indicate a connection between farms 6 and 7, this hypothesis could not be substantiated by epidemiological evidence. The fact that the time of ASFV introduction must have been at least two months before the confirmation of the disease, further increases the difficulty in identifying conclusive evidence for the route of virus introduction. The uncertainty of this estimate is therefore high.

### Outbreak farm 8 (Brandenburg)

#### Background information

The last outbreak reported from the federal state of Brandenburg so far was confirmed in February 2023. The farm was located in the south of the federal state in a restriction zone and close to outbreak farm 3. The wild boar population in the immediate vicinity was known to be ASFV-positive; the minimum distance from the farm to a positive wild boar was only 250 m.

At the time of the outbreak, the farm had 10 pigs and some sheep. It also harboured slaughtering facilities and a farm shop. The pigs were kept indoors in a stable without outdoor access, but biosecurity measures were implemented only at a minimal level.

#### Course of events

One pig that had died, tested positive for ASFV. After culling the remaining nine pigs, six tested positive for ASFV and none had ASFV-specific antibodies. This suggests that the virus was introduced only a few days prior to confirmation.

#### Epidemiological investigations

In the environment around the outbreak farm, the ASFV load was probably very high. It was thus hypothesized that ASFV was introduced indirectly, e.g. through a contaminated mechanical vector. Direct contact with wild boar was deemed unlikely, though it could not be entirely ruled out.

#### Genome sequencing

Full-length sequencing was initialized for samples from lung and spleen material of infected animals (2023ASP00680 – ct 22, 2023ASP00683 – ct 19). Sequencing libraries were constructed in duplicates and treated by hybridization capturing to increase the yield of ASFV-related reads (lib06119-22). In parallel, libraries were sent for external sequencing to further increase the amount of sequencing data (lib06111, lib06112). The combined data of all enriched libraries resulted in 19,803 ASFV reads that allowed for an initial assessment of the virus genome, but did not generate a complete genome. This number could be increased with the addition of the externally generated sequencing data, resulting in a total of 36,451 ASFV reads and the generation of a nearly complete virus genome (mean coverage = 28 reads/site). An alignment with the German reference sequence revealed the affiliation of the virus to Lineage IV, based on the identified marker mutation in the MGF 360-15R gene. Moreover, a 23-nucleotide long deletion could be observed in the intergenic region between MGF 360–13 L and MGF 360–14 L (g.32.788_32.810del) as well as substitutions in the EP424R gene (g.71.983 A > G), the M448R gene (g.80.770G > A), the I267L gene (g.171.436 C > T), one insertion in a homopolymer region (g.139.822insA) and nucleotide deletions in the MGF 360-21R (g.187.977_187.978del) and MGF 505-2R gene (g.34.454del) (Supplemental material Table S1). Genetic investigations in wild boar reported one ASFV-positive case with a nearly identical mutation pattern (missing the 23 nt-long deletion in the inter-genomic region) in close proximity to the affected facility (~ 20 km, 2022ASP03260, lib05997, Table 1 and Figure S1). Moreover, two more cases sharing most of these mutations were found in a similar distance in 2022 (20252022ASP03250, lib05995 and 2022ASP03254, lib05996, Table 1 and Figure S1), which provides evidence for a local circulation of a progenitor of the causative virus variant found on outbreak farm 8.

#### Conclusions

Considering the clinical course and the fact, that ASFV-positive wild boar infected with genetically closely related virus variants were found in the vicinity of the farm, an indirect or even direct introduction from wild boar seems plausible. The uncertainty of this estimate is low.

### Outbreak farm 9 (Mecklenburg-Western Pomerania)

#### Background information

In June 2024, an outbreak was reported from a fattening pig farm in the south of Mecklenburg-Western Pomerania. The farm was surrounded by agricultural land and completely fenced. At the time of the outbreak, 3,577 pigs were kept on the farm.

In Mecklenburg-Western Pomerania, the last ASF cases in wild boar were reported in 2022. In addition, these cases occurred approx. 200 km west from the outbreak farm. Apart from two guard dogs, there were no other animals on the farm. On the farm, no bedding was used and the six structurally identical stables with two completely separate compartments in each of the stables seemed to be in a good and hygienic state.

#### Course of events

Four days after pigs kept in the same compartment showed clinical signs, they were treated with antibiotics due to a suspected bacterial infection. After the body temperature in the treated animals stayed high, the responsible veterinarian euthanized two diseased pigs and submitted the carcasses for necropsy. The suspicion of ASF was officially confirmed the same night. In total, only animals from three of 16 pens pens in a single compartment were affected.

In the frame of culling, 200 samples were taken (at least 15 per compartment) and tested for ASFV and ASFV-specific antibodies. Of the 200 samples analysed, 15 tested positive for ASFV, and nine of these also tested positive for ASFV-specific antibodies.

#### Epidemiological investigations

Virus introduction through pig movement was considered unlikely as pigs were only brought into the farm before the estimated time of virus introduction and placed into another compartment. Due to fencing, closed stables and the absence of virus in the surrounding wild boar population, direct but also indirect virus introduction through infected wild boar seemed unlikely. As the course of disease concentrated on a single compartment, vehicle traffic (mainly the delivery of feed) was considered improbable as a source of virus introduction. One staff member was an active hunter, but stated that the last hunting activity had taken place before the presumed time of virus introduction. Furthermore, the hygiene measures, which, according to the statements of the employees, were complied with, made an indirect virus introduction appear unlikely.

#### Genome sequencing

For outbreak farm 9, DNA was extracted from lymph nodes and spleen material and processed for library construction. To accelerate the initial data acquisition, libraries were prepared for nanopore sequencing and sequenced on a P2 solo platform. Highest read counts were obtained from sample 2024ASP01428 (ct 19) with a total of 2,239 ASFV reads (lib06609, mean coverage = 9 reads/site) that mapped to ASFV Germany 2020/21 (LR899193). In parallel, the external Illumina sequencing was initiated and yielded 541,829 ASFV reads for the same sample, resulting in a mean coverage of 423 reads/site. A detailed genomic analysis revealed thirteen differences between the assembled virus genome and the German reference from 2020: the three marker mutations of Lineage III (MGF 360–10 L, MGF 505-4R, MGF 100–3 L), five mutations in intergenic regions (2 deletions, 3 insertions), one synonymous as well as three nonsynonymous mutations in annotated genes and one insertion in the MGF 110–14 L gene (Supplemental material Table S1). Beside the detected indels, the synonymous mutation in gene C315R (g.88.242 A > G) and the two nonsynonymous mutations in the genes B962L (g.93.333 C > A) and CP2475L (g.124.296T > A) were reported for the first time and therefore did not provide further information to elucidate the origin of the new virus variant. However, the third non-synonymous mutation was located in the MGF 505-6R (g.41.409G > A) and was found before in cases of outbreak farm 6 and 7 as well as in wild boar cases in the northeast of Germany in 2022 and 2024. These findings point towards a repeated introduction of MGF 505-6R virus variants from the northeast border region between Germany and Poland, like it was described for the other ASFV case in Mecklenburg-Western Pomerania (outbreak farm 4). Strikingly, the so far unique I329L mutation found in the ASFV variant from 2021, which became a characteristic for cases from Mecklenburg-Western Pomerania, could not be detected in the virus variant from outbreak farm 9, which was also shown by the segregation of both outbreak sequences in different phylogenetic groups in the Lineage III cluster (Figure S1).

#### Conclusions

The epidemiological investigations failed to identify the route of virus introduction, but some potential routes such as direct introduction through infected wild boar, could be ruled out. However, evidence of a genetic relationship between viruses from outbreak farm 9 and cases observed in the same region could be found in the occurrence of a common MGF 505-4R mutation, suggesting regional transmission. The uncertainty of this estimate is high.

In Table 2 basic data of all nine outbreaks are summarised.

**Table 2:** Summarised basic data of the nine ASF-outbreaks.

Outbreak farm	Federal state	Date of official disease confirmation	Husbandry system	Approx. no. of pigs on farm	Virus lineage ^20^	Outbreak hypothesis	Estimated time period between virus introduction and detection
1	Brandenburg	15.07. 2021	Backyard farm	2	Lineage III	Infected wild boar population in close vicinity = > indirect introduction through contaminated material	5–10 days
2	Brandenburg	15.07. 2021	Backyard farm	4	Lineage III	Infected wild boar population in close vicinity = > indirect introduction through contaminated material	4–7 days
3	Brandenburg	15.07. 2021	Organic breeding farm	300	Lineage IV	Infected wild boar population in close vicinity = > indirect introduction through contaminated material	4–7 days
4	Mecklenburg-Western Pomerania	15.11.2021	Fattening farm	4,000	Lineage III	Point introduction through human activity	5–7 days
5	Baden- Wuerttemberg	25.05.2022	Organic fatting farm	19	None	Point introduction through human activity	5–7 days
6	Lower Saxony	02.07.2022	Breeding farm	765	Lineage III	Point introduction through human activity/trade	7–14 days
7	Brandenburg	02.07.2022	Fattening farm	1,038	Lineage III	Point introduction through human activity	2–3 months
8	Brandenburg	28.02.2023	Hobby farm	10	Lineage IV	Infected wild boar population in close vicinity = > indirect introduction through contaminated material	4–7 days
9	Mecklenburg-Western Pomerania	05.06.2024	Fattening farm	3,577	Lineage III	Point introduction through human activity	7–10 days

## Discussion

In the present paper, ASF outbreaks in German domestic pig holdings from 2021 to 2024 are described. Outbreaks in Hesse and Rhineland-Palatinate from summer 2024 were excluded. These outbreaks represent a separate cluster and the circumstances differ somewhat from the other outbreaks (with many outbreaks occurring within a short period and in close proximity), including this information here would go beyond the scope of the present study. However, a separate publication on these cases is planned. In coherence with outbreaks in other countries^12–14,16,28^, biosecurity measures and human activities seem to have played a major role in the German ASF outbreaks. This could be observed in small farms where very obvious biosecurity deficiencies have been identified (e.g. outbreak 1 and 2) or in large commercial and apparently well-managed farms where one must assume that a possibly tiny biosecurity gap has led to the introduction of a virus (e.g. outbreak 4 and 6). Human behaviour and attitudes towards the consistent and, in particular, rigorous implementation of biosecurity measures are complex and depend on a variety of factors^29–31^. Also, it seems to be human nature that routine activities may lead to negligence that often remains without consequences. Under unfavourable circumstances, however, minor deviations from the defined procedures may have far-reaching consequences, such as an animal disease outbreak. In addition to the compliance with and the consequent implementation of biosecurity measures, human behaviour also plays a crucial role in the success of epidemiological investigations. The investigators often have to rely on the accuracy and truth of statements made by the involved farm staff. Usually, it can or should be assumed that the statements made correspond to the truth. In the case of a known infringement against legal regulations, it must also be considered that at least parts of the truth may be concealed, making it impossible for the investigator to draw the right and final conclusions^32^. This is obviously a major problem and complicates epidemiological investigations and thus effective disease control. However, the reasons for concealing the truth are complex and are certainly not only based on a personal fear of admitting mistakes. There are also economic and social consequences that may follow. In the event of a proven offense, farmers may receive only reduced compensation, which may increase their financial losses^33^. Furthermore, it can be assumed that their social standing among farming colleagues might suffer, as it could be assumed that they might have put other farms at risk, by provoking further disease outbreaks^34^. This can happen directly through transmission to another farm, but also indirectly by farms suffering from the restrictions resulting from a disease outbreak on another farm.

Moreover, the accuracy of reported information may decrease with the time between pathogen introduction and the confirmation of the disease outbreak. This may apply to some of the ASFV outbreaks reported here, since in some cases, the time of introduction was probably several weeks before the detection of ASF on the affected premises.

The vast majority of outbreaks occurred in late spring or summer, which is consistent with observations from other European countries. It is hypothesized that this pattern may be due to increased contact with potentially contaminated materials (such as fresh grass, clothing, and machinery) and is often linked to the disease dynamics in wild boar populations^10,35,36^.

It is notable that symptomatic treatment with suspected bacterial infection occurred in several farms with only delayed testing for ASFV. This is particularly surprising in areas, where ASF was already present or near in the wild boar population at the time of the outbreak (e.g. outbreak 1, 2 and 7). On the one hand, this behaviour is problematic with regards to a potential spread of the virus, either directly through contaminated needles or indirectly through contaminated mechanical vectors, like clothes. On the other hand, it also harbours the risk that the detection of the disease and the corresponding measures will be delayed and that the disease can spread unhindered, e.g. through animal movements. These experiences emphasize the need for large-scale awareness campaigns, not only at the beginning of an epidemic but constantly and easily accessible for all affected groups of persons^37,38^. As a consequence, unsuccessful treatment of pigs with antimicrobials should be immediately followed by sampling the animals and testing for ASFV in countries, where ASF is present.

Furthermore, the relatively slow spread of the virus within herds on the outbreak farms is also striking and confirms that ASF is not a highly contagious disease in many settings^1^. In five out of the nine cases, it was observed that only one unit was affected or additional units, in which already diseased animals were relocated. However, in holdings where all pigs of the farm were kept together (as in outbreaks 1, 2 and 5), all pigs were infected, suggesting a relatively rapid spread or a virus source to which all animals had access. At farms, where the animals were clearly separated, usually in conventional pig holdings (like in outbreak 4, 6,7 and 9), often only one or two compartments were affected. That indicates a rather slow transmission between units. This might be related to the virus characteristics with blood being the most effective vehicle and the fact that transmission via blood is still rather rare^36,39^. However, good internal biosecurity measures could also prevent a fast virus spread between the separate compartments (like in outbreak 7). These observations were also presented in other outbreak descriptions^14,37,40^.

It has been described several times in the past that infected wild boar play a role in domestic pig outbreaks^1,10,12,41^. For the German outbreaks, where farms were located in areas with an infected wild boar population, indirect contact to ASFV-infected wild boar was usually the most likely hypothesis for ASFV introduction. However, particularly for outbreaks that have occurred outside of Brandenburg, an association with infected wild boar was considered unlikely. Considering the surveillance effort in the wild boar population, it has to be mentioned that the sensitivity of the surveillance system is often not high enough to detect circulating virus in a wild boar population at a low prevalence level. Schulz, et al.^42^ showed in a study with Estonian ASF surveillance data that considering the sample sizes, a virus circulation would have not been detected if the assumed prevalence was lower than 2%. However, as described in the above-mentioned study, it can be assumed that a circulating virus in a wild boar population necessarily leads to an increased mortality in wild boar, which would almost certainly be noticed after some time^42^. No ASF-positive cases in wild boar were detected subsequently to the German outbreaks on farms located far away from the restriction zones I and II. This suggests that an introduction through direct wild boar contact was highly unlikely and that an infection in wild boar has not been missed. However, this does not rule out the possibility of indirect virus introduction through contaminated material that has been in contact with infected wild boar. Testing not only for ASFV, but also for ASFV-specific antibodies in domestic pig outbreaks, is not done on a regular basis. However, particularly in outbreak 7, where ASFV-specific antibodies and simultaneously negative results for ASFV were observed, which suggests an introduction several weeks before disease detection, the usefulness of performing both investigations was demonstrated.

The conclusions and the extend of tracing back and forward differ significantly depending on the knowledge of these laboratory results, thus increasing the chance to avoid further spread from either the original virus source or from the first and potentially also from subsequent affected farms.

Summarizing the epidemiological findings, it is often extremely difficult if not impossible to determine the cause of virus introduction precisely. That was also described by other colleagues^10,12,41^. Epidemiologists are often left with hypotheses, supported by varying levels of certainty, that must be accepted by both investigators and the authorities implementing disease control measures. However, new techniques allowing to examine virus genomes in detail have become an indispensable complement to routine diagnostics by offering the possibility to assess genetic relationships between known and emerging virus variants. For ASF, NGS of virus genomes supports epidemiological investigations by identifying and tracking characteristic mutations observed in wild boar and domestic pig populations. Based on these genetic markers, sequencing can provide information about the most likely origin of ASFV variants and enables a genomic epidemiology, as it was shown before that the occurrence of variant-specific marker correlates with the geographic dispersion of ASFV variants in Germany^20^. This conclusion is further supported by the results of this study, as the majority of the herein reported viruses from outbreaks in domestic pig holdings were genetically closely related to virus variants circulating in wild boar populations from the same geographic region, encompassing all outbreak farms in Brandenburg (1, 2, 3, 7 and 8) as well as in Mecklenburg-Western Pomerania (4 and 9). In contrast, two introductions of ASFV into facilities distantly located from known outbreak areas were observed (outbreak farm 5 - Baden-Wuerttemberg and 6 - Lower Saxony), which could be distinguished into two different scenarios on the basis of the generated whole genomes: an introduction of ASFV from a, most likely, German outbreak with a high genetic similarity to an active outbreak in another ASFV-affected pig holding (outbreak farm 6) and the incursion of an ASFV variant from an external source, missing key genetic markers that were found in all German virus variants at that time (outbreak farm 5). Despite the many advantages of NGS, it is important to acknowledge that both, the results and their interpretation, have inherent limitations. At present, the availability of sequence data remains limited, which increases the risk of drawing misleading conclusions. For instance, while the detection of a similar sequence in another outbreak may suggest that the same variant occurred at both holdings, it is equally possible that this sequence is circulating in other regions where it has simply not yet been detected or sequenced.

The description of German ASF outbreaks in domestic pigs may help to identify starting points to decrease the probability of virus introduction. Biosecurity measures that are consequently maintained, trained employees and regular awareness campaigns to all involved groups of persons are crucial. It is understandable that frustration or even resignation arises, when farms that have seemingly done everything right are still affected. This can give the impression that farmers are powerless against virus introduction. Yet, this impression may be wrong. High standards of biosafety with regular audits, trainings, discussions and the closing of gaps may reduce the risk of an ASF outbreak substantially and it is all the more important to constantly raise awareness and motivate affected groups of people not to flag their efforts to protect their farms and their animals.

## Supplementary Information

Below is the link to the electronic supplementary material.

Supplementary Material 1

## Data Availability

The original data used for the analyses can be obtained from the author after approval by the responsible institutions of the affected federal states. The assembled whole-genomes were submitted to the European Nucleotide Archive (ENA, European Bioinformatics Institute, Hinxton, UK) under the project accession PRJEB89459.

## References

[1] Oļševskis, E. et al. African swine fever virus introduction into the EU in 2014: experience of Latvia. *Res. Vet. Sci.* **105**, 28–30. 10.1016/j.rvsc.2016.01.006 (2016). 27033903 10.1016/j.rvsc.2016.01.006

[2] Smietanka, K. et al. African swine fever Epidemic, Poland, 2014–2015. *Emerg. Infect. Dis.* **22**, 1201–1207. 10.3201/eid2207.151708 (2016). 27314611 10.3201/eid2207.151708 PMC4918169

[3] Nurmoja, I. et al. Development of African swine fever epidemic among wild Boar in Estonia - two different areas in the epidemiological focus. *Sci. Rep.* **7**, 12562. 10.1038/s41598-017-12952-w (2017). 28970577 10.1038/s41598-017-12952-w PMC5624900

[4] Pautienius, A. et al. Prevalence and Spatiotemporal distribution of African swine fever in Lithuania, 2014–2017. *Virol. J.*10.1186/s12985-018-1090-8 (2018). 30454055 10.1186/s12985-018-1090-8 PMC6245807

[5] World Organisation for Animal Health. African swine fever (ASF) – Situation report 30. https://www.woah.org/app/uploads/2023/03/asf-report30.pdf (2023).

[6] Sauter-Louis, C. et al. Joining the club: first detection of African swine fever in wild Boar in Germany. *Transbound. Emerg. Dis.* **00**, 1–9. 10.1111/tbed.13890 (2020). 10.1111/tbed.13890 33085828

[7] Cadenas-Fernández, E., Ito, S., Aguilar-Vega, C., Sánchez-Vizcaíno, J. M. & Bosch, J. The role of the wild Boar spreading African swine fever virus in asia: another underestimated problem. *Front. Veterinary Sci.*10.3389/fvets.2022.844209 (2022). 10.3389/fvets.2022.844209 PMC9093143 35573420

[8] Jori, F. et al. *In Understanding and Combatting African Swine Fever - A European Perspective* 197–228 (Wageningen Academic, 2021).

[9] European Food Safety Authority. African swine fever and outdoor farming of pigs. *EFSA J.* **19**, e06639. 10.2903/j.efsa.2021.6639 (2021). 34140998 10.2903/j.efsa.2021.6639 PMC8188572

[10] Nurmoja, I. et al. Epidemiological analysis of the 2015–2017 African swine fever outbreaks in Estonia. *Prev. Vet. Med.*10.1016/j.prevetmed.2018.10.001 (2018). 30482617 10.1016/j.prevetmed.2018.10.001

[11] Gogin, A., Gerasimov, V., Malogolovkin, A. & Kolbasov, D. African swine fever in the North Caucasus region and the Russian federation in years 2007–2012. *Virus Res.* **173**, 198–203. 10.1016/j.virusres.2012.12.007 (2013). 23266725 10.1016/j.virusres.2012.12.007

[12] Boklund, A. et al. Risk factors for African swine fever incursion in Romanian domestic farms during 2019. *Sci. Rep.* **10**, 10215. 10.1038/s41598-020-66381-3 (2020). 32576841 10.1038/s41598-020-66381-3 PMC7311386

[13] Lamberga, K., Serzants, M. & Ojsevskis, E. African swine fever outbreak investigations in a large commercial pig farm in latvia: a case report. *Berl Munch. Tierarztl. Wochenschr*. **132**, 151–155. 10.2376/0005-9366-18031 (2019).

[14] Zani, L. et al. African swine fever in a Bulgarian backyard Farm—A case report. *Veterinary Sci.* **6**, 94 (2019). 10.3390/vetsci6040094 PMC6958451 31766581

[15] Vergne, T., Gogin, A. & Pfeiffer, D. U. Statistical exploration of local transmission routes for African swine fever in pigs in the Russian Federation, 2007–2014. *Transbound. Emerg. Dis.* **64**, 504–512. 10.1111/tbed.12391 (2017). 26192820 10.1111/tbed.12391

[16] Malakauskas, A. et al. African swine fever outbreaks in Lithuanian domestic pigs in 2019. *Anim. (Basel)*. 10.3390/ani12010115 (2022). 10.3390/ani12010115 PMC8749716 35011221

[17] Bergmann, H. et al. Identification of risk factors for African swine fever: A systematic review. *Viruses* **14**, 2107 (2022). 36298662 10.3390/v14102107 PMC9611626

[18] Bellini, S., Casadei, G., De Lorenzi, G. & Tamba, M. A. Review of risk factors of African swine fever incursion in pig farming within the European Union scenario. *Pathogens*10.3390/pathogens10010084 (2021). 10.3390/pathogens10010084 PMC7835761 33478169

[19] Rusinà, A. et al. Semi-Quantitative risk assessment of African swine fever virus introduction in outdoor pig farms. *Pathogens* **12**, 709 (2023). 37242379 10.3390/pathogens12050709 PMC10222819

[20] Forth, J. H. et al. African swine fever virus – variants on the rise. *Emerg. Microb. Infect.*10.1080/22221751.2022.2146537 (2022). 10.1080/22221751.2022.2146537 PMC9793911 36356059

[21] Carrascosa, A. L., Bustos, M. J. & de Leon, P. Methods for growing and titrating African swine fever virus: field and laboratory samples. *Curr. Protoc. Cell. Biol.*10.1002/0471143030.cb2614s53 (2011). 22161547 10.1002/0471143030.cb2614s53

[22] Li, H. Minimap2: pairwise alignment for nucleotide sequences. *Bioinformatics* **34**, 3094–3100. 10.1093/bioinformatics/bty191 (2018). 29750242 10.1093/bioinformatics/bty191 PMC6137996

[23] den Dunnen, J. T. et al. HGVS recommendations for the description of sequence variants: 2016 update. *Hum. Mutat.* **37**, 564–569. 10.1002/humu.22981 (2016). 26931183 10.1002/humu.22981

[24] Camacho, C. et al. BLAST+: architecture and applications. *BMC Bioinform.* **10**, 421. 10.1186/1471-2105-10-421 (2009). 10.1186/1471-2105-10-421 PMC2803857 20003500

[25] Wylezich, C. et al. Next-generation diagnostics: virus capture facilitates a sensitive viral diagnosis for epizootic and zoonotic pathogens including SARS-CoV-2. *Microbiome* **9** (51). 10.1186/s40168-020-00973-z (2021). 10.1186/s40168-020-00973-z PMC7896545 33610182

[26] Giammarioli, M. et al. Complete genome of African swine fever virus genotype II in central Italy. *Microbiol. Resour. Announc*. **12**, e0136422. 10.1128/mra.01364-22 (2023). 37166310 10.1128/mra.01364-22 PMC10281112

[27] Bundesministerium für Ernährung und Landwirtschaft. *African swine fever in Germany*. https://food.ec.europa.eu/system/files/2022-07/reg-com_ahw_20220707_asf_deu.pdf (2022).

[28] Viltrop, A., Reimus, K., Niine, T. & Mõtus, K. Biosecurity levels and farm characteristics of African swine fever outbreak and unaffected farms in Estonia; What can be learned from them? *Animals* **12**, 68 (2022). 10.3390/ani12010068 PMC8749753 35011174

[29] Mankad, A. Psychological influences on biosecurity control and farmer decision-making. A review. *Agron. Sustain. Dev.* **36**10.1007/s13593-016-0375-9 (2016).

[30] Bucini, G. et al. Connecting livestock disease dynamics to human learning and biosecurity decisions. *Front. Vet. Sci.*10.3389/fvets.2022.1067364 (2023). 10.3389/fvets.2022.1067364 PMC9896627 36744225

[31] Racicot, M., Venne, D., Durivage, A. & Vaillancourt, J. P. Evaluation of the relationship between personality traits, experience, education and biosecurity compliance on poultry farms in Québec, Canada. *Prev. Vet. Med.* **103**, 201–207. 10.1016/j.prevetmed.2011.08.011 (2012). 21940059 10.1016/j.prevetmed.2011.08.011

[32] World Organisation for Animal Health. A field manual for animal disease outbreak investigation and management. (2018).

[33] Gerdes, U. (2024).

[34] Mankad, A. & Curnock, M. Emergence of social groups after a biosecurity incursion. *Agron. Sustain. Dev.* **38**10.1007/s13593-018-0520-8 (2018).

[35] Rogoll, L. et al. Seasonal occurrence of African swine fever in wild Boar and domestic pigs in EU member States. *Viruses* **15**, 1955 (2023). 37766361 10.3390/v15091955 PMC10536336

[36] European Food Safety Authority. Epidemiological analyses of African swine fever in the European union (November 2017 until November 2018). *EFSA J.* **16**, e05494. 10.2903/j.efsa.2018.5494 (2018). 32625771 10.2903/j.efsa.2018.5494 PMC7009685

[37] Li, Y. et al. African swine fever in a commercial pig farm: outbreak investigation and an approach for identifying the source of infection. *Transbound. Emerg. Dis.* **67**, 2564–2578. 10.1111/tbed.13603 (2020). 32419367 10.1111/tbed.13603

[38] Guinat, C., Wall, B., Dixon, L. & Pfeiffer, D. U. English pig farmers’ knowledge and behaviour towards African swine fever suspicion and reporting. *PLoS One*. 10.1371/journal.pone.0161431 (2016). 27684556 10.1371/journal.pone.0161431 PMC5042443

[39] Schulz, K., Conraths, F. J., Blome, S., Staubach, C. & Sauter-Louis, C. African swine fever: fast and furious or slow and steady? *Viruses-Basel*10.3390/v11090866 (2019). 10.3390/v11090866 PMC6783890 31533266

[40] Ardelean, F. et al. The course of African swine fever in Romanian backyard holdings - A case report. *Veterinary Med. Sci.* **7**, 2273–2279. 10.1002/vms3.592 (2021). 10.1002/vms3.592 PMC8604127 34378334

[41] Kim, H. J. et al. Outbreak of African swine fever in South Korea. *Transbound. Emerg. Dis.* 67, 473–475 10.1111/tbed.13483 (2020). 10.1111/tbed.13483 31955520

[42] Schulz, K. et al. African swine fever re-emerging in estonia: the role of seropositive wild Boar from an epidemiological perspective. *Viruses* **13**, 2121 (2021). 34834928 10.3390/v13112121 PMC8625046

