# Supplementary material for: African swine fever outbreaks in German pig holdings – experiences, epidemiological considerations and genome sequences
Source: Sci Rep. 2026 Jan 29;16:4350. doi: 10.1038/s41598-026-36441-1 (PMC12864975; doi:10.1038/s41598-026-36441-1)
Supplement: Supplementary file 1 — Supplementary Material 1 [file 41598_2026_36441_MOESM1_ESM.docx]

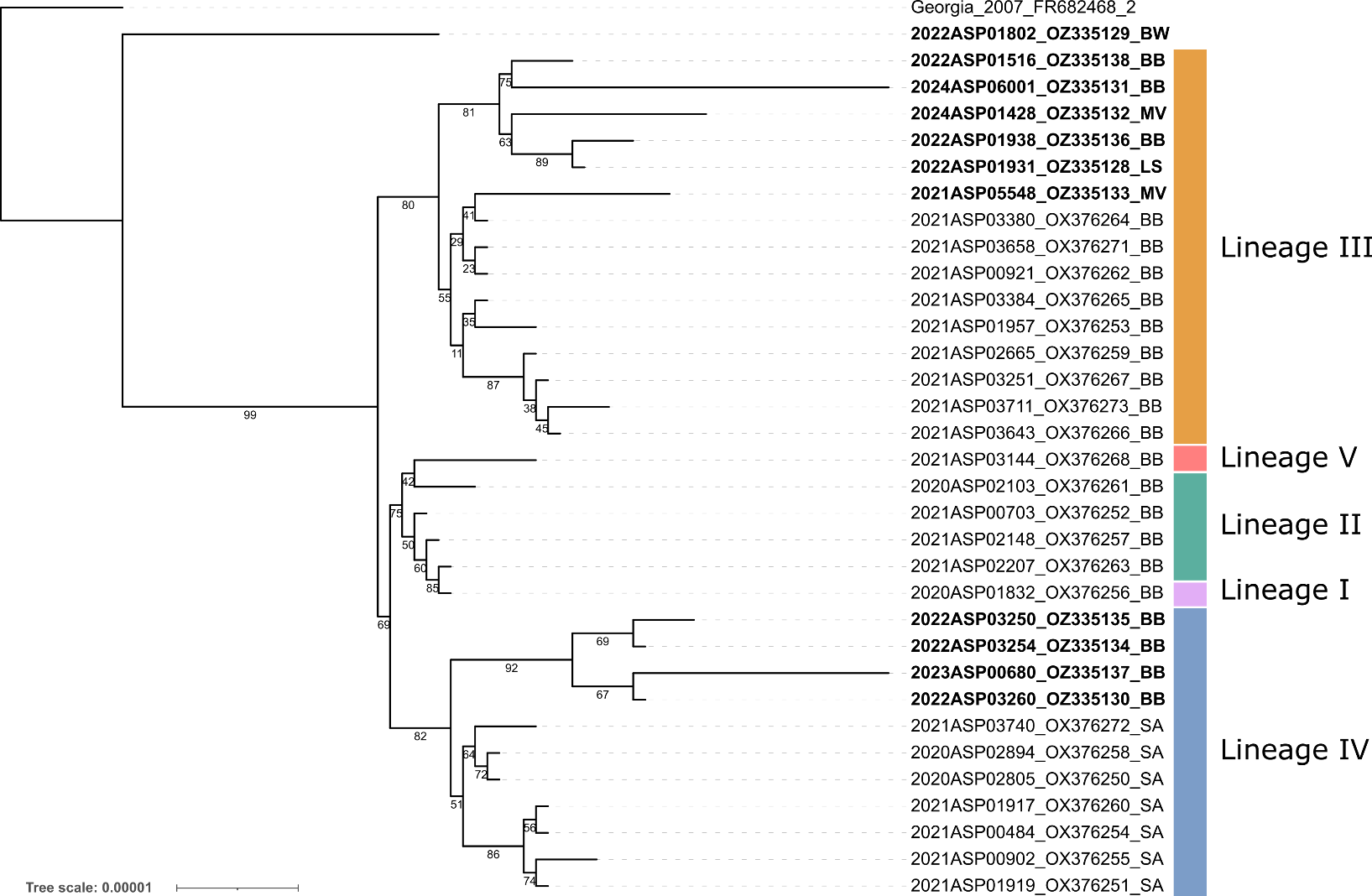


**Figure S1** Maximum-Likelihood phylogenetic tree of published German ASFV variants with Georgia 2007/1 (FR682468.2) serving as an outgroup. Sequences generated in this study are highlighted in bold. The phylogenetic tree was constructed with IQTree (v.3.0) with 100.000 ultrafast bootstraps and enabled ModelFinder^1^ feature (best fit model: F81 + F). Visualization and annotation of the tree was conducted with iTOL v.7.2.2 (European Molecular Biology Laboratory)^2^ and Inkscape (v. 0.92). BW: Baden-Wuerttemberg; BB: Brandenburg; MV: Mecklenburg-Western Pomerania; LS: Lower Saxony; SA: Saxony.

**Table S2** Overview of identified genetic changes between the German reference (LR899193) and ASFV virus genomes obtained from the investigated outbreak farms. *Mutation positions are given in reference to the Georgia 2007 ASFV genome FR682468.2

| Lineage | | | Lineage specific mutations (compared to LR899193) | Position * | Affected Gene | Mutation type | Impact on amino acid sequence |
| --- | --- | --- | --- | --- | --- | --- | --- |
| III | | | G > A | 27.197 | MGF 360-10L | Nonsense | Frameshift |
|  |  |  | -A | 37.027 | MGF 505-4R | Deletion | Frameshift |
|  |  |  | -A | 181.407 | MGF 100-3L | Deletion | Frameshift |
| IV | | | C > T | 50.922 | MGF 360-15R | Nonsense | Frameshift |
| Outbreak Farm | **Lib-No.** | **Lineage (according to Forth et al. 2022)** | **Non-Lineage specific mutations (compared to LR899193)** | **Position *** | **Affected Gene** | **Mutation type** | **Impact on amino acid sequence** |
| 1,2 | lib05117 | III | - | - | - | - | - |
| 3 | lib05058 + lib05059 | IV | - | - | - | - | - |
| 4 | lib05327 | III | T > A | 173.628 | I329L | Nonsynonymous | His293Leu |
| 5 | lib05910 | None | +CCCCC | 1.390 | Intergenic region | Insertion | None |
|  |  |  | -T | 2.963 | Intergenic region | Deletion | None |
|  |  |  | A > C | 9.107 | MGF 110-4L | Nonsynonymous | Asn65Lys |
|  |  |  | A > G | 10.668 | MGF 110-7L | Synonymous | None |
|  |  |  | +CC | 14.237 | MGF 110-14L | Insertion | Frameshift |
|  |  |  | -CCCCC | 15.682 | MGF 110-13Lb | Deletion | Frameshift |
|  |  |  | +GG | 17.632 | Intergenic region | Insertion | None |
|  |  |  | -G | 17.846 | Intergenic region | Deletion | None |
|  |  |  | +GGGGGG | 20.008 | Intergenic region | Insertion | None |
|  |  |  | +GGGGGG | 21.805 | Intergenic region | Insertion | None |
|  |  |  | +T | 22.898 | Intergenic region | Insertion | None |
|  |  |  | T > C | 26.425 | MGF 360-10L | Nonsynonymous | Asn |
|  |  |  | G > A | 35.753 | Intergenic region | Substitution | None |
|  |  |  | A > G | 39.308 | MGF 505-5R | Nonsynonymous | Ile330Val |
|  |  |  | A > G | 64.397 | K205R | Synonymous | None |
|  |  |  | A > C | 66.154 | K145R | Nonsynonymous | Tyr145Ser |
|  |  |  | +G | 103.312 | Intergenic region | Insertion | None |
|  |  |  | -TTTTTCAGTAGTGA | 129.276 | O174L | Insertion | Prolongation of ORF |
|  |  |  | C > G | 167.204 | E199L | Nonsynonymous | Ala85Pro |
|  |  |  | +T | 173.025 | Intergenic region | Insertion | None |
|  |  |  | C > A | 186.066 | ASFV G ACD 01940 | Nonsynonymous | Leu16Phe |
|  |  |  | -A | 187.997 | MGF 360-21R | Deletion | None |
|  |  |  | +A | 190.135 | DP60R | Insertion | Prolongation of ORF |
| 6 | lib05777 | III | T >A | 17.599 | Intergenic region | Base exchange | None |
|  |  |  | +A | 24.994 | Intergenic region | Insertion | None |
|  |  |  | G > A | 41.409 | MGF 505-6R | Nonsynonymous | Cys465Tyr |
|  |  |  | +A | 187.978 | Intergenic region | Insertion | None |
| 7 | lib05778 | III | T >A | 17.599 | Intergenic region | Base exchange | None |
|  |  |  | +A | 24.994 | Intergenic region | Insertion | None |
|  |  |  | G > A | 41.409 | MGF 505-6R | Nonsynonymous | Cys465Tyr |
|  |  |  | G > T | 72.358 | EP424R | Nonsynonymous | Gly255Cys |
| 8 | lib06111-12 +  lib06119-22 | IV | - AAGCAGTCTATAAGACAGTCTAT | 32.788 - 32.810 | Intergenic | Deletion (-23 nt) | None |
|  |  |  | -A | 34.454 | MGF 505-2R | Deletion | Frameshift |
|  |  |  | A > G | 71.983 | EP424R | Nonsynonymous | Asn130Asp |
|  |  |  | G > A | 80.770 | M448R | Synonymous | None |
|  |  |  | +A | 139.822 | Intergenic | Insertion | None |
|  |  |  | C > T | 171.436 | I267L | Synonymous | None |
|  |  |  | -AA | 187.977 – 187.978 | MGF 360-21R | Deletion | None |
| 9 | lib06605 (Illumina) + lib06609 (Nanopore) | III | +CCCCCC | 14.237 | MGF 110-14L | Insertion | +GlyGly |
|  |  |  | -C | 17.846 | Intergenic region | Deletion | None |
|  |  |  | +GGGGG | 21.805 | Intergenic region | Insertion | None |
|  |  |  | +A | 24.994 | Intergenic region | Insertion | None |
|  |  |  | +GTTATAATTAC | 26.252 | Intergenic region | Insertion (Tandem repeat) | None |
|  |  |  | G > A | 41.409 | MGF 505-6R | Nonsynonymous | Cys465Tyr |
|  |  |  | A > G | 88.242 | C315R | Synonymous | None |
|  |  |  | C > A | 93.300 | B962L | Nonsynonymous | Asp893Tyr |
|  |  |  | T > A | 124.296 | CP2475L | Nonsynonymous | Thr462Ser |
|  |  |  | -T | 173.455 | Intergenic region | Deletion | None |

References

**1** Kalyaanamoorthy, S., Minh, B., Wong, T. et al. ModelFinder: fast model selection for accurate phylogenetic estimates. Nat Methods 14, 587–589 (2017). https://doi.org/10.1038/nmeth.4285

**2** Ciccarelli FD, Doerks T, von Mering C, Creevey CJ, Snel B, Bork P. Toward automatic reconstruction of a highly resolved tree of life. Science. 2006 Mar 3;311(5765):1283-7. doi: 10.1126/science.1123061. Erratum in: Science. 2006 May 5;312(5774):697. PMID: 16513982.
